# The Metabolism of Baicalin in Rat and the Biological Activities of the Metabolites

**DOI:** 10.1155/2012/404529

**Published:** 2012-05-13

**Authors:** Yi Wang, Jingyu Yang, Xian Li, Jinhui Wang

**Affiliations:** ^1^Department of Natural Products Chemistry, Shenyang Pharmaceutical University, Liaoing 110016, China; ^2^Key Laboratory of Marine Drugs, Chinese Ministry of Education and School of Medicine and Pharmacy, Ocean University of China, Qingdao 266003, China

## Abstract

Baicalin is one of the major bioactive constituents of Scutellariae Radix, but the biotransformation of it is poorly understood. In this paper, the metabolism of baicalin in rat was studied. Nine metabolites including one new compound were isolated and identified structurally. The plausible scheme for the biotransformation pathways of baicalin in the rats was deduced. And the main metabolites were evaluated for their antioxidation and anti-inflammation biological activities for the first time.

## 1. Introduction

Scutellariae Radix, the root of *Scutellariae baicalensis *Georgi, is widely used in traditional Chinese formulations. Baicalin (baicalein 7-*O*-glucuronide) is one of the major bioactive constituents of it, which possesses antiallergic, anti-inflammatory activity and antioxidation and has been used for the treatment of hepatitis, hyperlipidemia, and lipolysis [1].

 It is well known that the process of drug metabolism affects therapeutic effects of drug. The biotransformation of baicalin is poorly understood, and that is due in part to difficulties that have been encountered in obtaining enough amounts to identify the structure of the metabolites and study the bioactivities of them. Although some works on the metabolism of baicalin have been investigated with the development of chromatography-spectrographic technology, because of the lack of metabolites quantitatively, many questions about the biological activities of the metabolites still remained after administration [2–5]. To gain additional insight into its metabolism and the biological activities of the metabolites, we isolated the metabolites from urine and feces of rats and identified their structures on the basis of physicochemical properties and spectroscopic data analysis. Nine metabolites including one new compound were obtained. At the same time, the antioxidation and anti-inflammation biological activities of the metabolites were investigated. It is the first time that the metabolism and the bioactivities of the metabolites of baicalin were studied comprehensively.

## 2. Materials and Methods

### 2.1. Drugs

Baicalin was isolated and purified from Scutellariae Radix according to the method reported previously [6]. The crude drug was further purified with open ODS column, and the purity of the material exceeded 95% that was confirmed by HPLC.

### 2.2. Dosing Procedure

 Seven-week old Wistar rats (five males and five females), weighing 180 to 210 g, were used for the experiments. For isolation of metabolites, the rats were orally administered baicalin (500 mg/Kg) suspended in 0.5% CMC-Na solution with repeated dosing three times, then urine and feces were obtained by using a metabolic cage for 48 h. All samples were stored below −20°C until use.

### 2.3. Isolation of Metabolites

Urine samples from rats were combined, filtrated, and adjusted to PH 4 with HCl and performed by chromatographic separation on a resin column eluting with water (part I), C_2_H_5_OH/H_2_O (3 : 7) (part II), and C_2_H_5_OH/H_2_O (9 : 1) (part III). Part III was subjected to ODS column eluting with CH_3_CN/H_2_O gradiently (from 95% H_2_O to 65% H_2_O) to obtain the compounds **M1**(45 mg), **M2**(10 mg), **M3**(5 mg), and **M4**(3 mg). Preparative HPLC on ODS column was used to further purify 70% H_2_O fraction from ODS column with CH_3_OH/CH_3_CN/H_2_O (20 : 20 : 60) to obtain compounds **M7**(2 mg) and **M9**(3 mg), and 65% H_2_O fraction from ODS column was also chromatographed on preparative HPLC with CH_3_OH/H_2_O (70 : 60) to obtain compounds **M6**(3 mg) and **M8**(3 mg). Preparative TLC on silica gel was used to further purify 75% H_2_O fraction from ODS column to obtain compound **M5**(25 mg).

 Feces samples from rats were combined, suspended in the water and adjusted to PH 7 with NaHCO_3_-saturated aqueous solution, and then filtrated. The filtrate was adjusted to PH 4 with HCl and subjected to a resin column eluting with water (part I), C_2_H_5_OH-H_2_O (3 : 7) (part II), and C_2_H_5_OH-H_2_O (9 : 1) (part III). Then part II and part III were combined and subjected to Sephadex LH-20 eluting with C_2_H_5_OH-H_2_O (from 100% H_2_O to 30% H_2_O): C_2_H_5_OH-H_2_O (2 : 8) fraction from LH-20 gave **M1**(20 mg); C_2_H_5_OH-H_2_O (35 : 65) fraction gave **M5**(32 mg); C_2_H_5_OH-H_2_O (4 : 6) fraction was further purified by preparative HPLC on an ODS column eluting with CH_3_OH-H_2_O (2 : 8) and gave **M6**(2 mg) and **M8**(2 mg).

### 2.4. Identification of Baicalin and Metabolites

#### 2.4.1. General Experimental Procedures

 Semipreparative high-performance liquid chromatography was performed on an ODS column (YMC-pack ODS-A, 10 × 250 mm, 5 *μ*m) at a flow rate of 3 mL/min. Analyses of compounds by electrospray ionization mass (ESI-MS) spectroscopy were conducted with a Thermo-Finnigan LCQ quadrupole ion-trap mass spectrometer (Thermo, San Jose, CA). Nuclear magnetic resonance (NMR) spectroscopy was performed on a BRUKER-APX-300 to obtain ^1^H (300 MHz) and ^13^C (75 MHz) spectra of the pigment in dimethyl sulfoxided-6 (DMSO-d6).

#### 2.4.2. Metabolites Data

**Figure 1:**
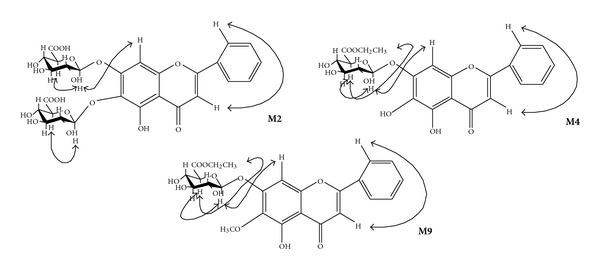
The NOE correlations on the NOESY spectra of metabolites **M2**, **M4**, and **M9**.

**M2:** yellow powder. ESI-MS m/z: 645.1 [M+Na]^+^, 623.1 [M+H]^+^, 621.1 [M-H]^−^, 621.1 [M-H]^−^, 445.0 [M-glcA-H]^−^. ^1^H-NMR (300 MHz, DMSO-d_6_): *δ* 7.61 (2H, m, H-3′,5′), 7.60 (1H, m, H-4′), 8.10 (2H, d, *J* = 6.0 Hz, H-2′,6′)], 7.12 (1H, s, H-8), 7.07 (1H, s, H-3), 12.89 (1H, s, HO-5), 5.27 (1H, d, *J* = 7.4 Hz, 7-glcA-1), 4.97 (1H, d, *J* = 7.3 Hz, 6-glcA-1), 4.00 (1H, d, *J* = 9.5 Hz, 7-glcA-5), 3.57 (1H, d, *J* = 9.6 Hz, 6-glcA-5), 3.1–3.4 (m). NOESY: given as in Figure 1.

**M3:** yellow powder. ESI-MS m/z: 483.1 [M+Na]^+^, 459.0 [M-H]^−^, 445.0 [M-CH_3_-H]^−^. ^1^H-NMR (300 MHz, DMSO-d_6_): *δ* 7.62 (2H, m, H-3′,5′), 7.60 (1H, m, H-4′), 8.08 (2H, d, *J* = 6.0 Hz, H-2′,6′)], 7.06 (1H, s, H-8), 7.02 (1H, s, H-3), 12.57(1H, s, HO-5), 8.29 (1H, s, HO-6), 5.29 (1H, d, *J* = 6.9 Hz, 7-glcA-1), 4.23 (1H, d, *J* = 9.3 Hz, 7-glcA-5), 3.1–3.4 (m).

**Figure 2:**
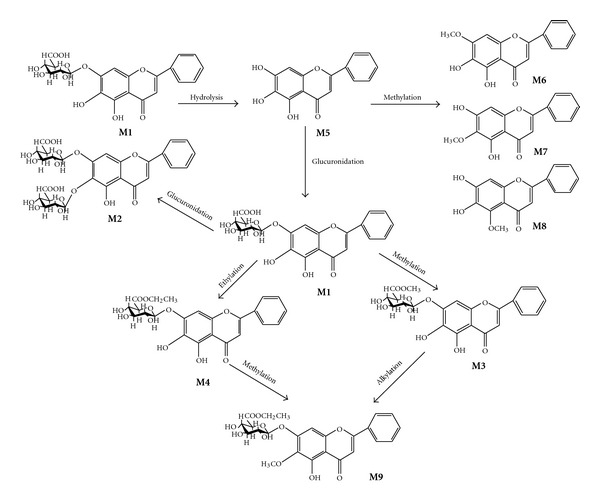
The plausible scheme for the biotransformation pathways of baicalin in the rats.

**M4:** yellow powder. ESI-MS m/z: 497.1 [M+Na]^+^, 475.1 [M+H]^+^, 473.0 [M-H]^−^, 445.0 [M-CH_3_CH_2_-H]. ^1^H-NMR (300 MHz, DMSO-d_6_): *δ* 7.60 (2H, m, H-3′,5′), 7.59 (1H, m, H-4′), 8.08 (2H, d, *J* = 6.0 Hz, H-2′,6′), 7.07 (1H, s, H-8), 7.02 (1H, s, H-3), 12.59 (1H, s, HO-5), 8.65 (1H, s, HO-6). 5.28 (1H, d, *J* = 7.2 Hz, 7-glcA-1), 4.18 (1H, d, *J* = 9.6 Hz, 7-glcA-5), 3.1–3.4 (m), 1.21 (3H, t, *J* = 7.2 Hz, CH_3_), 4.11 (2H, m, CH_2_). NOESY: given as in Figure 2.

**M6:** yellow powder. ESI-MS m/z: 307.0 [M+Na]^+^, 282.9 [M-H]^−^. ^1^H-NMR (300 MHz, DMSO-d_6_): *δ* 7.61 (2H, m, H-3′,5′), 7.61 (1H, m, H-4′), 8.07 (2H, d, *J* = 6.0 Hz, H-2′,6′), 6.25 (1H, s, H-8), 6.96 (1H, s, H-3), 12.51 (1H, s, HO-5), 8.4 (1H, s, HO-6), 3.80 (3H, s, OCH_3_).

**M7:** yellow powder. ESI-MS m/z: 874.8 [3 M+Na]^+^, 590.9 [2 M+Na]^+^, 307.0 [M+Na]^+^, 282.9 [M-H]^−^. ^1^H-NMR (300 MHz, DMSO-d_6_): *δ* 7.63 (2H, m, H-3′,5′), 7.62 (1H, m, H-4′), 8.07 (2H, m, H-2′,6′), 6.32 (1H, s, H-8), 7.02 (1H, s, H-3), 12.51 (1H, s, HO-5), 10.8 (1H, s, HO-7), 3.86 (3H, s, OCH_3_).

**M8:** yellow powder. ESI-MS m/z: 307.1 [M+Na]^+^, 282.9 [M-H]^−^. ^1^H-NMR (300 MHz, DMSO-d_6_): *δ* 7.57 (2H, m, H-3′,5′), 7.57 (1H, m, H-4′), 8.37 (2H, m, H-2′,6′), 6.54 (1H, s, H-8), 6.19 (1H, s, H-3), 8.4 (1H, s, HO-6), 3.72 (3H, s, OCH_3_).

**Figure 3:**
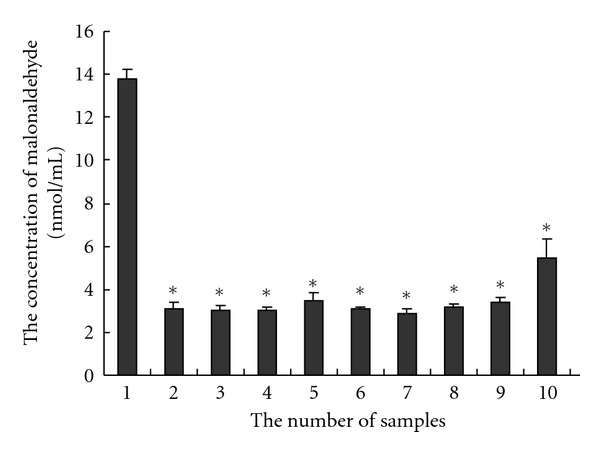
The effects of baicalin (**M1**) and the metabolites baicalein (**M5**) and baicalein 6,7-di-O-*β*-glucopyranoside (**M2**) on liver lipid peroxide of rats. (1, saline; 2, extract of ginkgo biloba leaves injection 250 mg/mL; 3, **M1** 40 mg/mL; 4, **M1** 20 mg/mL; 5, **M1** 10 mg/mL; 6, **M5** 20 mg/mL; 7, **M5** 10 mg/mL; 8, **M5** 5 mg/mL; 9, **M2** 40 mg/mL; 10, **M2** 20 mg/mL; 11, **M2** 10 mg/mL; *, *P* < 0.01  *t*-test compared with saline group).

**M9:** yellow powder. ESI-MS m/z: 511.1 [M+Na]+, 523.0 [M+Cl]−, 487.0 [M-H]−. ^1^H-NMR (300 MHz, DMSO-d6): *δ* 7.63(2H, m, H-3′,5′), 7.62 (1H, m, H-4′), 8.09 (2H, m, H-2′,6′), *δ* 7.13 (1H, s, H-3), 7.07 (1H, s, H-8), 12.83 (1H, s, HO-5). *δ* 5.36 (1H, d, *J* = 6.9 Hz, 7-glcA-1), 4.18 (1H, d, *J* = 9.6 Hz, 7-glcA-5), 3.1–3.4 (m). 3.78 (3H, s, OCH3), 1.20 (3H, t, *J* = 6.6 Hz, CH3), and 4.14, 4.11 (each 1H, dd, *J* = 15.0, 6.6 Hz, CH2O). NOESY: given as in Figure 3.

### 2.5. Biological Activities of the Metabolites

#### 2.5.1. Antioxidation

Livers were obtained from rats and disposed of the blood. Twenty g of the livers was divided into pieces and prepared into homogenate with physiological salt solution by refiner about 8000 r/min and then added physiological salt solution to 400 mL to be 5% tissue homogenate. Saline was used for blank; extract of ginkgo biloba leaves injection 250 mg/mL was used for positive control; baicalin (**M1**) and the metabolites baicalein (**M5**) and baicalein 6,7-di-*O*-**β**-glucopyranuronoside (**M2**) were also analyzed.

 All the numbered tubes were added with 1.5 mL 5% tissue homogenate, reference substance flavonoids extracts of ginkgo, and metabolites of baicalin. Negative control was physiological saline. All the tubes were incubated at 37°C for 1 h, and 1.5 mL 20% trichloroacetic acid was added. All the tubes were mixed, and standing for 10 min, centrifuged at 3000 rpm for 10 min. The supernatant was added with 0.67% thiobarbituric acid and heated in boring water for 10 min. The cool solution was tested on spectrophotometer at 532 nm to obtain data. Based on the standard curve of malonaldehyde bis(diethyl acetal), malonaldehyde (nmol/mL) was calculated by multiplying parameter 68.89.

#### 2.5.2. Effect on LPS-Induced NO Yielding

 Wistar rats (weight 200 to 250 g) were used for experiments. The rats were decapitated and treated with celiac injection with 15 mL RPMI1640 culture medium. Irrigating solution was centrifuged at 1000 r/min for 10 min. The supernatant was discarded. The deposition was suspended in RPMI1640 culture medium (10% FBS) and incubated for 1 h, and nonadherent cell is discarded. Cells on the wall were washed by RPMI1640 culture medium (10% FBS), dyed by nigrosine, and counted on blood counting chamber. The cell density was adjusted to 60 × 10^4^ cells/cm^2^. The cells were incubated overnight. The culture medium was changed to serum-free medium. LPS (lipopolysaccharide), different metabolites of baicalin, and L-NAME (NO synthase inhibitor) were added. After 24 h of incubation, the contents of NO_2_^−^ in medium were tested with Griess colorimetry. Experimental results were analyzed statistically with SPSS software. L-NAME was used for positive control; the metabolites, baicalein 6,7-di-*O*-**β**-glucopyranuronoside (**M2**), baicalein 7-*O-*β**-D-glucopyranuronoside methyl ester (**M3**), baicalein (**M5**), and 7-*O-*methyl-baicalein (**M6**), were also analyzed.

## 3. Results and Discussion

### 3.1. The Metabolism of Baicalin in Rats

Baicalin was orally administered to rats. The collected urine and feces samples were extracted and analyzed as described in experimental part. In addition to baicalin, a total of 8 metabolites including baicalin (**M1**), baicalein 6,7-di-*O-*β**-D-glucopyranuronoside (**M2**), baicalein 7-*O-*β**-D-glucopyranuronoside methyl ester (**M3**), baicalein 7-*O-*β**-D-glucopyranuronoside ethyl ester (**M4**), baicalein (**M5**), 7-*O-*methyl-baicalein (**M6**), 6-*O-*methyl-baicalein (**M7**), 5-*O-*methyl-baicalein (**M8**), 6-*O-*methyl-baicalein-7-*O-*β**-D-glucopyranuronoside ethyl ester (**M9**) were found present in urine and 4 metabolites (**M1**, **M5**, **M6**, and **M8**) in feces. The structures of metabolites were elucidated by a combination analysis of their chromatographic behavior, analysis of MS, ^1^HNMR data, NOESY data, and spectral comparison to several reference substances.

***Metabolites* *M1 and M5***

Compared with reference substances,** M1** and **M5** have the same chromatographic behavior and NMR data. So **M1** and **M5** were identified as baicalin and baicalein, respectively.

**Metabolites M2**

Compared with baicalin, there are two groups of the data of glucuronide on the ^1^HNMR spectrum of **M2** [*δ*: 5.27 (1H, d, *J* = 7.4 Hz, 7-glcA-1), 4.97 (1H, d, *J* = 7.3 Hz, 6-glcA-1), 4.00 (1H, d, *J* = 9.5 Hz, 7-glcA-5), 3.57 (1H, d, *J* = 9.6 Hz, 6-glcA-5), 3.1–3.4 (m)], and the chemical shift of *δ* 8.6 (1H, s, HO-6) disappeared, but *δ* 12.56 (1H, s, HO-5) still can be observed. At the same time combined with NOESY spectrum (Figure 1), the correlation between the anomeric proton of glucuronide (*δ* 5.27) and the proton of position 8 (*δ* 7.12) was observed. All these showed that there were two glucuronides at the position of C-6 and C-7. On the ESI-MS, the m/z: 645.1 [M+Na]^+^, 623.1 [M+H]^+^, 621.1 [M-H]^−^, 621.1 [M-H]^−^, 445.0 [M-glcA-H]^−^ was accordant. The chemical structure of **M2** was determined as baicalein 6, 7-di-*O-*β**-glucopyranuronoside.

***Metabolites* *M3* and *M4***

Compared with the ^1^HNMR spectrum of baicalin, **M3** revealed considerable similarity that there was one group of the data of glucuronide but differed in one proton of methyl *δ* 3.61 (3H, s), and the chemical shift of the proton at position 5 of the glucuronide was shifted to downfield by 0.6, which suggested the existence of glucopyranuronoside methyl ester. So **M3** was determined as baicalein 7-*O-*β**-D-glucopyranuronoside methyl ester. On the ESI-MS, the m/z: 483.1 [M+Na]^+^, 459.0 [M-H]^−^, 445.0 [M-CH_3_-H]^−^ was accordant. **M4** showed very similar ^1^HNMR spectrum with** M3**, except one group of ethyl data [*δ*: 1.21 (3H, t, *J* = 7.2 Hz, CH_3_), 4.11 (2H, m, CH_2_)]. Combined with NOESY spectrum (Figure 1), there was a correlation between the anomeric proton of one glucuronide (*δ* 5.28) and the proton of position 8 (*δ* 7.07), so the positions of glucuronide can be determined that was position 7. The correlation between the proton at position 5 of the glucuronide (*δ* 4.18) and the methyl proton (*δ* 1.21) was also observed. Thus, the structure of **M4** was determined as baicalein 7-*O-*β**-D-glucopyranuronoside ethyl ester.

**Metabolites M6, M7, and M8**

Compared with the ^1^HNMR spectrum of baicalin, there was no glucuronide signal but one methyl proton in these three metabolites. The molecular weight of them (m/z: 307.1 [M+Na]^+^, 282.9 [M-H]^−^) also showed m/z 14 more than baicalin. All of them showed A2A'B2 coupled system for ring-C without any substituent that was the same as baicalin. On the ^1^HNMR spectrum of** M6**, OH-5 and OH-6 still can be observed, but OH-7 disappeared, and one OCH_3_ was observed, and at the same time the chemical shift of the proton of position 8 was shifted to high field by 0.4, compared with baicalein (**M5**). Therefore, the chemical structure of **M6** was determined as 7-*O-*methyl-baicalein. Compared with **M5**, *δ* 12.51(1H, s, HO-5) and *δ* 10.8 (1H, s, HO-7) still can be observed, but the proton signal of HO-6 was substituted by *δ* 3.86 (3H, s, OCH_3_-6) on **M7**. The chemical structure of **M7** was determined as 6-*O-*methyl-baicalein compared with reference [7]. **M8** had the different retention characteristic with **M6** and **M7**. On the ^1^HNMR spectrum of **M8**, the proton signal of HO-5 was substituted by *δ* 3.72 (3H, s, OCH_3_), and the chemical shift of the proton of position 8 shifted to high field by 0.3, so the chemical structure of **M8** was determined as 5-*O-*methyl-baicalein.

**Metabolite M9**

Compared with baicalin, **M9** revealed considerable similarity, but differences were that one group of ethoxy data [*δ* 1.20 (3H, t, *J* = 6.6 Hz, CH_3_) and *δ* 4.14, 4.11 (each 1H, dd, *J* = 15.0, 6.6 Hz, CH_2_O)], one methyl proton (*δ* 3.78 (3H, s, CH3)), but the active proton of OH at position 6 disappeared. The chemical shift of the proton at position 5 of the glucuronide shifted to downfield by 0.5, and the proton at position 5 of glucuronide (*δ*4.18) had a correlation with the methyl proton of oxyethyl group (*δ* 1.20) combined with NOESY spectrum (Figure 1), so this metabolite can be determined as one glucopyranuronoside ethyl ester. On NOESY, the correlation between the anomeric proton of the glucuronide (*δ* 5.36) and the proton of position 8 (*δ* 7.07) was observed, so the positions of glucuronide can be determined to be position 7, and the methyl proton was at position 6. On the ESI-MS, the m/z: 511.1 [M+Na]^+^, 523.0 [M+Cl]^−^, 487.0 [M-H]^−^ was also accordant. Thus, the chemical structure of **M9 **was determined as 6-*O-*methyl-baicalein-7-*O-*β**-D-glucopyranuronoside ethyl ester. To our knowledge, this compound was the first time to be isolated and identified.

Based on the structures of these metabolites, a plausible scheme for the biotransformation pathways of baicalin in the rats was shown in Figure 2. The results of the present study demonstrated that the major metabolites of baicalin were baicalin, baicalein, and glucuronide after oral administration, and at the same time, small amount of alkylated products were also found, which was a little different from [4]. This may be for the more alkylation reacted on glucuronides and aglycone with longer time. The baicalin is very difficult to be absorbed into blood directly [8]. In the gastrointestinal tracts of rats, baicalein was hydrolyzed into baicalein by **β**-glucuronidase produced by intestinal bacteria. Thus, baicalein can be easily absorbed into blood. Through enterohepatic circulation, baicalein was transformed to glucuronides and then modified to kinds of alkylated glucuronides to demonstrate bioactivities.

### 3.2. The Biological Activities of the Metabolites

The effects of baicalin (**M1**) and the metabolites (**M5**), (**M2**) on liver lipid peroxide of rats were studied as shown in Figure 3. As can be seen, baicalin (**M1**), baicalein (**M5**), and baicalein 6,7-di-*O-*β**-glucopyranuronoside (**M2**) have more stronger antioxidation than positive control, flavonoids extract of ginkgo. Baicalein (**M5**) was even much stronger.

**Figure 4:**
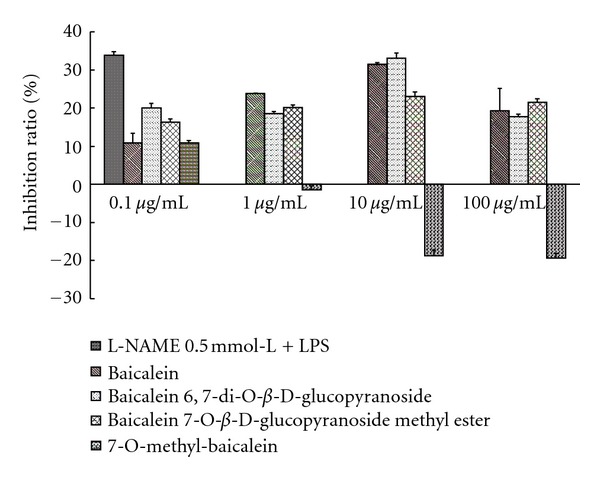
The inhibition of metabolites on LPS-induced NO yielding.

Figure 4 showed the effect of the metabolites (**M5**), (**M2**), (**M3**), and (**M6**) on NO yielded by macrophage. As can be seen, LPS (10 *μ*g/mL) induced macrophage to yield NO in 24 h, and positive control L-NAME can inhibit NO yielding significantly. Metabolites baicalein (**M5**) and baicalein 6,7-di-*O-*β**-glucopyranuronoside (**M2**) had significantly inhibiting effect on NO yielding induced by LPS within the range of the test content. Baicalein 7-*O-*β**-D-glucopyranuronoside methyl ester (**M3**) and 7-*O-*methyl-baicalein (**M6**) had no inhibiting effect on NO yielding induced by LPS within the range of the test content.

As described above, baicalin was metabolized to baicalein, glucuronide, and methylated products though metabolism of intestinal bacteria and enterohepatic circulation. Although studies on the metabolism of drugs have been investigated with the development of LC-MS, the bioactivities on metabolites are still lacking of investigation. The results of bioactive experiments of these metabolites in our experiments demonstrated that baicalein and glucuronide showed significant potential on antioxidation and affections on LPS-induced NO yielding compared with reference substance. However, when substituents were replaced by alkyl, bioactivity was depressed. To our knowledge, this is the first time that the metabolites of baicalin were investigated on the antioxidation and anti-inflammation biological activities. These results suggested though baicalein had good biological availability, it could not be used for its instability, while glucuronides of baicalein will be the perspective lead compounds for their good stability and bioactivities.
